# Massive venous air embolism with bleeding caused by femoral vein injury during total hip arthroplasty: A case report

**DOI:** 10.1097/MD.0000000000023614

**Published:** 2021-01-29

**Authors:** Ji Young Min, Kyungmoon Roh, Seunghee Cho, Sanghyun Hong, Mee Young Chung

**Affiliations:** a Department of Anesthesiology and Pain Medicine, Eunpyeong St. Mary's Hospital, College of Medicine, The Catholic University of Korea, Seoul; b Department of Anesthesiology and Pain Medicine, Incheon St. Mary's Hospital, College of Medicine, The Catholic University of Korea, Incheon, Republic of Korea.

**Keywords:** total hip arthroplasty, transesophageal echocardiography, vascular injury, venous air embolism

## Abstract

**Introduction:**

Venous air embolism (VAE) from vascular injuries, is of rare occurrence but can result in catastrophic complications during total hip arthroplasty (THA). Early recognition and prompt management of vascular injury are required to avoid severe complications. Especially, bleeding is generally associated with profound hypotension in venous injury. We report an unusual complication of venous air embolism induced by femoral vein rupture during THA.

**Patient concerns:**

A 54-year-old male patient with a history of old left acetabular fracture was scheduled for THA. We experienced massive bleeding and VAE induced by femoral vein rupture during total hip arthroplasty. The BP suddenly dropped from 100/70 mm Hg to 80/50 mm Hg with massive bleeding. ETCO_2_ and SaO_2_ decreased profoundly.

**Diagnosis:**

The VAE was diagnosed by the change in end- tidal CO_2_ (ETCO_2_) and change of vital signs, so we performed ABGA and inserted TEE for confirmination.

**Interventions:**

For treatment, patient was managed by oxygen therapy, inotropics, vasopressor, transfusion and surgical repair.

**Outcomes:**

Upon consulting with a cardiologist, the patient was extubated the next day and was transferred to the general ward and recovered without serious complications. He stayed for 17 days until finally discharged without complications

**Conclusion:**

Preoperative vascular imaging may be recommended in the revisional case of THA or in patients with the history of hip trauma. The monitoring of ETCO_2_ and TEE might be helpful to recognize VAE earlier and therefore to avoid catastrophic complications through adequate treatment.

## 1 Introduction

Nowadays total hip arthroplasty (THA) surgery is considered a well-established procedure and safe and vascular injuries during THA are rare. The overall reported incidence is 0.1% to 0.3% of surgery,^[1]^ but the occurrence can be serious with high mortality risk. The incidence of a pulmonary embolism on vascular injuries during THA is 1.6%.^[2]^ The common iatrogenic causes reported are acetabular penetration, reaming and screw fixation, extraction of the acetabular component, femoral cerclage wire and inappropriate placement of anterior acetabular retractors and scalpel.^[3]^ We report an unusual complication of venous air embolism induced by femoral vein rupture during THA.

## 2 Case description

A 54-year-old male patient with a history of old left acetabular fracture was scheduled for THA. He was on medication for hypertension and was severely obese with body weight 88.2 kg, height 165.5 cm and body mass index of 32.2. He had persistent pain in the left hip area and was recently diagnosed with left traumatic OA. In the operating room, the vital signs were blood pressure 120/90 mm Hg, heart rate 68 per minute, and SpO_2_ 97%. Subsequently, the patient was intubated after injecting propofol 200 mg and succinylcholine 100 mg, after which rocuronium 50 mg was injected for muscle relaxation. The anesthesia was maintained with desflurane, N_2_O and O_2_. The patient was put in the right lateral position, and routine monitoring (electrocardiogram, blood pressure [BP], SpO_2_, bispectral index, esophageal temperature and end-tidal CO_2_ [ETCO_2_]) was maintained throughout the surgery.

About 40 minutes of initiation of surgery, massive bleeding occurred from the femoral vein. The femoral vein was injured while dissecting the severe fibrotic adhesion around the hip joint, which had apparently formed after the past accident. The BP suddenly dropped from 100/70 mm Hg to 80/50 mm Hg with massive bleeding. The bleeding site was packed with saline gauze by an orthopedic surgeon. Colloid solution and packed red cell (PRC) were administered in accordance with the estimated blood loss, followed by ephedrine 10 mg twice and atropine 0.5 mg, but the BP was not recovered. A right radial arterial catheter was inserted for the monitoring of blood pressure and arterial blood gas analysis (ABGA). The heart rate (68 beat per minute) was recovered but the BP (75/54 mm Hg) stayed below normal. Phenylephrine 100 μg was injected 3 times. PRC transfusion was continued while dopamine (2–5 μg/kg/minutes), dobutamine (5–10μg/kg/minutes) and epinephrine (0.03–0.1 μg/kg/minutes) were infused continuously.

**Figure 1:**
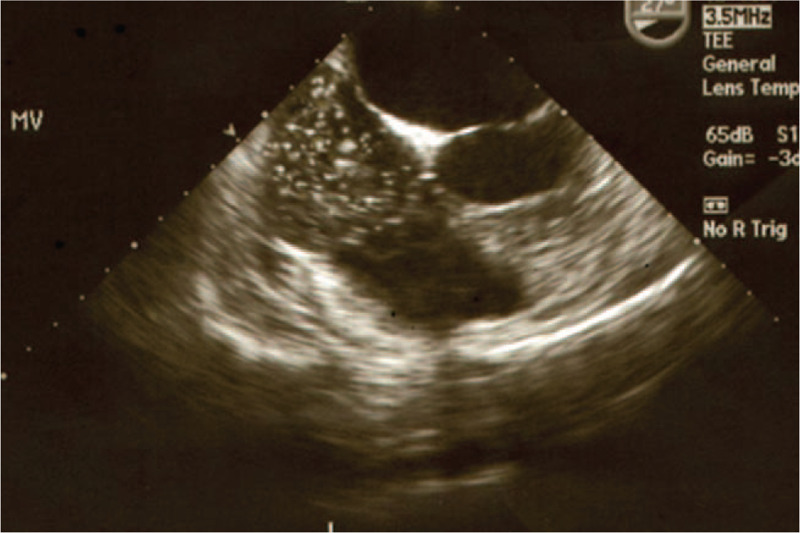
Many air bubbles flow into right atrium on TEE.

**Figure 2:**
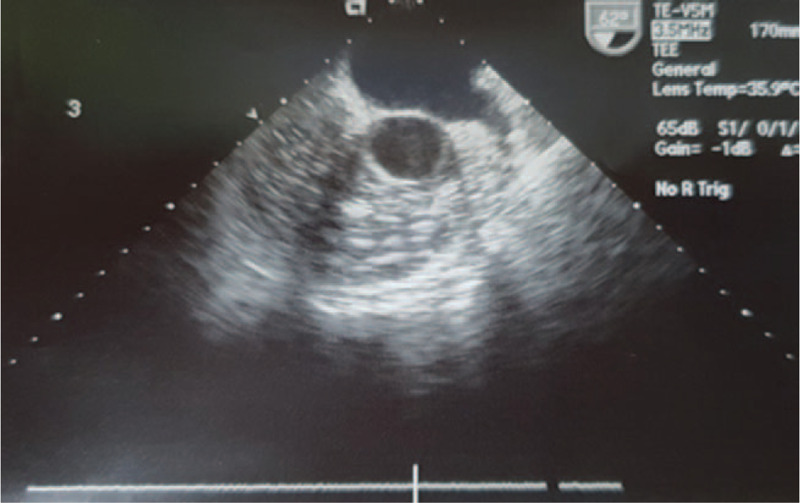
Many air bubbles flow into right ventricle and pulmonary artery on TEE.

Nevertheless, BP stayed around 70/40 mm Hg and heart rate was around 50 per minute. ETCO_2_ decreased from 29 mm Hg to 20 mm Hg, and then again to 18 mm Hg, while SaO_2_ decreased to 93%. At that time, ABGA was pH 7.248, PaCO_2_ 56.3 mm Hg, PaO_2_ 74.2 mm Hg, B.E –3.7, and SaO_2_ 94.2% on FiO2 1.0. Under the strong impression of the occurrence of air embolism, N_2_O was discontinued while FiO_2_ was turned to 1.0, and the left hip was placed lower than the hearts position. Transesophageal echocardiography (TEE) was promptly monitored. The real-time image of TEE acquired while the vascular surgeon was repairing the ruptured femoral vein revealed a continuous influx of air into the right atrium and pulmonary artery (Figs. 1 and 2). After the completion of vein clamping, the influx of air seemed to markedly slow down on TEE and the vital signs were gradually stabilized. ABGA was pH 7.286, PaCO2 48.8 mm Hg, PaO2 111.9 mm Hg, B.E –4.1, and SaO2 97.8% on FiO2 1.0. During the operation, plasma solution 4000 ml, normal saline 1050 ml, colloid 1500 ml, PRC 14 U and fresh frozen plasma 5 U were administered. Blood loss was estimated to be about 6000 ml by external observation of suction bottle, operation field, floor, blood-soaked gauzes and so on and urine output was 200 ml. The duration of the surgery was 3 hours 45 minutes and anesthesia was performed for 4 hours 30 minutes.

Upon consulting with a cardiologist, the patient was extubated the next day and was transferred to the general ward, where he stayed for 17 days until finally discharged.

## 3 Discussion

Vascular injury during THA is associated with significant morbidity including loss of limb (15%), as well as mortality (7%).^[3]^ The most prevalent injured vessels during THA are the common femoral artery, the external iliac artery, the superior gluteal artery, the external iliac vein, and the common femoral vein in the order of frequency.^[2]^ The common femoral artery and vein are only separated from the capsule by iliopsoas, rendering these vessels at risk of damage from the placement of a retractor or by scalpel, especially during the anterolateral approach to the hip.^[4]^ It occurs frequently in female patients and on the left side in arthroplasty, which reflects the closer proximity of the vessels to the hip joint in these 2 categories.^[5]^ However, Shuai et al^[6]^ described that other factors such as the type of surgery (infection or revision) might be more important for the vascular injury. Abularrage et al^[7]^ reported an increase in the incidence of 0.04% in primary THA to 0.19% in revision surgery. In our case, fibrosis and severe adhesions had developed around the left hip joint after the traffic accident, possibly causing deformation in the anatomy of the femoral vein. As a result, this vessel was at high risk of injury. Some authors have recommended routine preoperative vascular imaging during revision surgery.^[8]^ On the retrospective evaluation of this case, we concluded that it would have been safer to carry out vascular imaging preoperatively.

In the present case, lacerations on the variated femoral vein caused heavy bleeding intraoperatively and were recognized immediately. Reported complications include death in 7.3%, amputation in 1.6%, persistent ischemia in 7.3%, stroke in 1.7%, and pulmonary embolism in 1.7% of patients.^[2]^ Especially, bleeding is generally associated with profound hypotension in venous injury.^[9]^

The occurrence of damage in the major vessel could be managed with surgical intervention and volume therapy in most of the cases.^[10]^ Although very rare, venous injury associated with the pulmonary embolism may induce disastrous complications. Early recognition is hypothesized to be of importance and prompt treatment for pulmonary embolism must be done when such an event occurs intraoperatively.^[11]^

End-tidal carbon dioxide (ETCO_2_) monitor is the most convenient and practical monitor used in the operating room.^[12]^ The separation phenomenon, an opposite trend in decreased ETCO2 and increased PaCO2 can be an indicator of pulmonary embolism.^[13]^ In addition to the separation phenomenon, we could confirm the flow of numerous air bubbles into the right atrium and pulmonary artery on TEE. We considered these signs as pulmonary air embolism. After the vein was clamped, the influx of air seemed to markedly slow down on TEE and the vital signs and ABGA were gradually stabilized. TEE is currently the most sensitive monitoring device for VAE, detecting as little as 0.02 ml/kg of air administered by bolus injection.^[11]^ The major deterrents to TEE are invasiveness and expensiveness, and TEE requires expertise and constant vigilance.^[12]^ TEE might be useful in the patients at higher risk of VAE, such as patients with history of hip trauma, or undergoing revision THA, because TEE is not affected by electrocautery and lateral positioning during THA and is the most sensitive monitoring device for VAE.^[13]^

The primary goal in the treatment of VAE is the prevention of further air entry and the reduction of the volume of air. The morbidity and mortality of VAE are directly related to the volume of air entrainment and the rate of accumulation.^[12]^ These variables are mainly determined by the position of the injured vein and the right side of the heart.^[12]^ We adjusted the left hip to be placed lower than the level of the right heart. If the presence of VAE is confirmed, N_2_O should be discontinued and the patient should be placed on 100% oxygen, which facilitates adequate tissue perfusion and reduces the size of the VAE.^[14]^

In conclusion, anesthetic management for THA may require vigilant monitoring. Especially, vascular anatomy should be confirmed by vascular imaging preoperatively in patients with a trauma history of the hip joint or revision THA. The monitoring of ETCO_2_, TEE, etc., might be helpful to recognize VAE at an early stage and avoid catastrophic complications through the period of adequate treatment.

## Author contributions

**Conceptualization:** Ji Young Min, Mee Young Chung.

**Software:** Kyungmoon Roh, Sanghyun Hong.

**Writing – original draft:** Ji Young Min, Kyungmoon Roh, Mee Young Chung.

**Writing – review & editing:** Ji Young Min, Seunghee Cho.

## Abbreviations

Abbreviations: ETCO_2_ = end-tidal CO_2_, THA = total hip arthroplasty, VAE = venous air embolism.
